# Cellular elements in the developing caecum of Japanese quail (*Coturnix coturnix japonica*): morphological, morphometrical, immunohistochemical and electron-microscopic studies

**DOI:** 10.1038/s41598-019-52335-x

**Published:** 2019-11-07

**Authors:** Aalaa M. AbuAli, Doaa M. Mokhtar, Reda A. Ali, Ekbal T. Wassif, K. E. H Abdalla

**Affiliations:** 1 Department of Zoology, Faculty of Science, Assiut University, Assiut, Egypt; 2 Department of Anatomy and Histology, Faculty of Vet. Medicine, Assiut University, Assiut, Egypt

**Keywords:** Cell biology, Anatomy

## Abstract

The present study aims to investigate the histological, histochemical and electron microscopic changes of the caecal proximal part of Japanese quail during both pre- and post-hatching periods starting from the 2^nd^ embryonic day (ED) until four weeks post-hatching. On the 2^nd^ and 3^rd^ ED, the primordia of caeca appeared as bilateral swelling on the wall of the hindgut. On the 7^th^ ED, the lamina propria/submucosa contained the primordia of glands. On the 8^th^ ED, rodlet cells could be observed amongst the epithelial cells. On the 9^th^ ED, the caeca began to divide into three parts with more developed layers. With age, the height and number of villi increased. On the 13^th^ ED, immature microfold cells (M-cells) could be identified between the surface epithelium of the villi. The caecal tonsils (CTs) appeared in the form of aggregations of lymphocytes, macrophages, dendritic cells and different types of leukocytes. Telocytes and crypts of Lieberkuhn were observed at this age. On hatching day, the crypts of Lieberkuhn were well-defined and formed of low columnar epithelium, goblet cells, and enteroendocrine cells. Post-hatching, the lumen was filled with villi that exhibited two forms: (1) tongue-shaped villi with tonsils and (2) finger-shaped ones without tonsils. The villi lining epithelium contained simple columnar cells with microvilli that were dispersed with many goblet cells, in addition to the presence of a high number of intra-epithelial lymphocytes and basophils. Moreover, the submucosa was infiltrated by numerous immune cells. CD3 immunomarker was expressed in intraepithelial lymphocytes, while CD20 immunomarker showed focal positivity in CTs. In conclusion, the caecal immune structures of quails at post-hatching were more developed than those in pre-hatching life. The high frequency of immune cells suggests that this proximal part may be a site for immunological surveillance in the quail caecum. The cellular organisation of the caecum and its relation to the immunity was discussed.

## Introduction

Padgett and Ivey^1^ were amongst the first to describe the development of *Coturnix coturnix japonica*. Zacchei^2^ analysed the quail embryo development and compared the time with a specific Hamburger Hamilton stage of chick development^3^.

Recently, the Japanese quail has been introduced as an ideal model for embryological studies^4^. In addition, quail has many advantages over other avian species as a suitable model for developmental biology studies, as summarised by Huss and colleagues^5^. Quails are small birds, easy to grow in a laboratory, they hatch in about 16 days, and they have a short lifespan^6,7^. Moreover, the quail has proven to be a model for the production of a transgenic avian^8,9^.

Some studies have demonstrated variations in the morphology and function of the avian alimentary tract, and these may be related to evolutionary events, type, and nature of food intake, and habitat^10,11^. The determination of the morphological features of the digestive system facilitates the alteration of the birds’ performance and maintains them in a healthy condition^12,13^.

The avian caeca are two blind end sacs that extend from the ileocaeco-colic junction. The caecum is diverse in its shape, size, and number amongst different avian species. Each consists of three parts: distal or apex, middle or body and proximal or base. The distal part is short and extended to the blind end, the long middle part possesses a wide lumen and a thin wall, and the short proximal part has a narrow lumen and a thicker wall. The mucosa is analogous to that of the small intestine, with fewer goblet cells and glands. The villi are well-developed at the proximal part, shorter in the middle part and either shorter or absent in the distal part^14,15^.

The caecal wall is thinner than other parts of the intestine, contains lymphatic tissues that are mostly found in the proximal part forming caecal tonsils (CTs). Kitagawa and colleagues^16^ revealed that CTs have an immune-defence role in the caecal environment through regulation of microflora proliferation in the caecum and prevention of the invasion of extracaecal microorganisms. This is performed by the organisation of large lymphatic nodules throughout the caecal mucosa forming CTs.

Many authors have discussed the roles of avian caeca; there is evidence showed that the caeca are involved in the microbial degradation of some carbohydrates^17,18^, absorption of nutrients and water^19^, microbial synthesis of vitamins^20^, digestion, and absorption of cholesterol^21^, and degradation of nitrogenous compounds^22^. The caeca are considered as sites for microbial fermentation of plant materials and are thought to play significant roles in the defence of the body against invasion by antigens^15,23^.

The purpose of the present study is to demonstrate the morphological features of caecum during the pre- and post-hatching periods of the development using light and electron microscopy, mainly focusing on the determination of the cellular components of the caecal wall and identifying their immunohistochemical and ultrastructural features.

## Materials and Methods

The current work was performed in accordance with the Egyptian laws and University guidelines for animal care. The National Ethical Committee of the Faculty of Veterinary Medicine, Assiut University, Egypt, has approved all the procedures in the present study.

### I-Sample collection

The samples employed in the present study consisted of 200 fertile Japanese quail eggs. In addition, 60 birds were sacrificed at 7^th^, 14^th^ days and 4 weeks post-hatching (20 birds at each sampling period). These samples were collected from the Faculty of Agriculture farm, Assiut University. The fertile eggs were incubated at a temperature of 37.8 °C and a relative humidity ranged from 55–65% throughout the incubation period. The eggs numbered and a random number generator used. Ten eggs were randomly selected from the incubator daily from the 2^nd^ to 15^th^ embryonic days (ED). Post-hatching, the birds were housed in cages in a temperature-controlled room (26 °C), natural light, food and water were supplied *ad libitum*. Standard food (dry matter: 45.1% starch, 22.7% crude protein, 12.3% ash, 4.6% lipids, 3.3% fibre, 3.9% sugar, 4% calcium, and 0.8% phosphate) were used for feeding of quail. On the 2^nd^ and 3^rd^ ED, the embryos were taken as a whole and from 5^th^ to 15^th^ ED, the abdominal cavities of quail embryos were opened and the caeca were excised.

### II-Histological and Histochemical analysis

Small specimens (1 × 1 × 0.5 cm) of the caeca at the 2^nd^ to 15^th^ embryonic days, hatching day, 7^th^, 14^th^ days and 4 weeks post-hatching were immediately fixed in Bouin’s fluid for 20 h. The fixed specimens were dehydrated in an ascending series of ethanol, cleared in methyl benzoate and embedded in paraffin wax. Serial transverse sections of 5–6 μm were stained using Harris Haematoxylin and Eosin^24^ and Crossmon’s Trichrome^25^. In addition, the sections were stained with PAS^26^ for demonstration of neutral carbohydrates and with Mercury bromophenol blue^27^ for determination of proteins.

### III-Immunohistochemical analysis

The immunohistochemistry was carried out on formalin-fixed caeca collected at the hatching day. The specimens were processed, embedded in paraffin, and the tissue sections were cut at 4μm. The sections were treated with 10 mL MoL Tris buffer and 1 mL MoL ethylene-diamine tetra-acetic acid, pH 9.0 for 20 min at 90 °C. The block of endogenous peroxidase was done by incubation the sections with 3% H2O2, followed by preincubation overnight at 4 °C in 1% bovine serum albumin in PBS. The sections were stained for 30 min at 37 °C, using the following antibodies that showed reactivity in avian species: a rabbit polyclonal anti-CD3 (1:200; Abcam, Cambridge, UK, ab828), a mouse polyclonal anti-CD20 (1:200; Abcam, Cambridge, UK, ab88247), a rabbit polyclonal anti-S100 protein antibody (1:200, Thermo Fisher Scientific, Cat. RB-044-A0), and a rabbit polyclonal anti-SIgA (1:400; Abcam, Cambridge, UK, ab2411), according to the method described by Hsu and colleagues^28^. The sections were counterstained with haematoxylin, and analysed using an Olympus microscope (Japan). In parallel, tissue specimens, in which the primary antibodies were omitted and replaced with buffer, served as negative controls.

### IV-Semithin sections and transmission electron microscopy (TEM)

Small specimens of the proximal part of the caecum at 8^th^, 13^th^ ED, hatching day, and two weeks post-hatching were fixed with 2.5% glutaraldehyde and 2.5% paraformaldehyde in 0.1 M sodium cacodylate buffer, pH 7.3 and left overnight at 4 °C. The samples were post-fixed in 1% osmic acid in 0.1 M sodium cacodylate for 2 h at room temperature, dehydrated in ethanol and embedded in Araldite-Epon mixture. Semithin sections (1 μm in thickness) were stained with toluidine blue and examined under a light microscope. Ultrathin sections were stained with uranyl acetate and lead citrate^29^ before examined under JEOL100 II transmission electron microscope.

### V-Scanning electron microscopy (SEM)

Small specimens of caecum at 8^th^, 13^th^ ED, hatching day, and 4 weeks post-hatching were fixed with 5% glutaraldehyde and 2.5% paraformaldehyde in 0.1 M sodium phosphate buffer, pH 7.3, at 4 °C for 24 h. They were post-fixed in 1% osmic acid in 0.1 M sodium phosphate for 2 h at room temperature, dehydrated by acetone and then were subjected to critical-point drying with a Polaron apparatus. Finally, they were coated with gold using JEOL-1100E ion-sputtering device and photographed with a JEOL scanning electron microscope.

### VI- Morphometrical measurements

Morphometrical studies were carried out on paraffin sections of the caeca during both pre- and post-hatching periods using Image–J software. The measurements were done on three sections of the caeca at each age from three samples. Different areas of caeca were measured as follows: the caecum diameter (μm), the thickness of muscle layer (μm), the villi height (μm), the epithelium thickness (μm), and the diameter of the lumen of the proximal part of the caecum (μm). All the data were presented by mean ± SE.

## Results

### Histological and histochemical analysis

**Figure 1:**
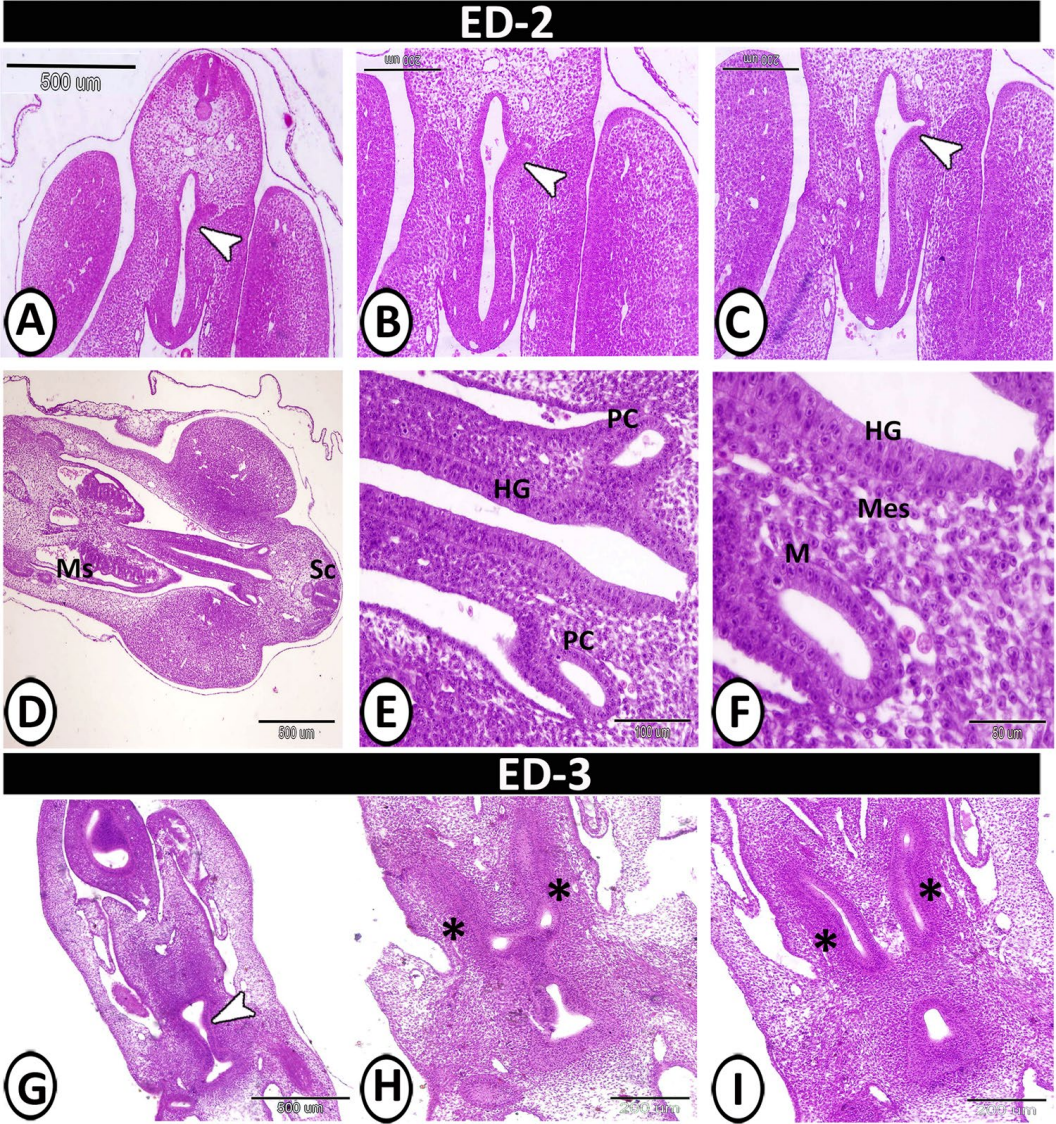
Transverse section (T.S) in the primitive hindgut on the 2^nd^ and 3^rd^ embryonic day (ED) stained by Haematoxylin and Eosin (H&E). (**A**,**B**) The beginning of invagination in the hindgut mucosa (arrowhead). (**C**) The complete invagination of caeca (arrowhead). (**D**) Primordia of caeca between mesonephros (Ms) and spinal cord (Sc). (**E**) Primordia of caeca (pC) between the hindgut wall (HG). (**F**) Hindgut mucosa (HG), undifferentiated mesenchymal tissue (Mes) and mucosa of caeca (M). (**G**) The hindgut tube showed invagination of caeca (arrowhead). (**H**) Partial separation of hindgut parts (asterisk). (**I**) The beginning of caecal tube formation (asterisk).

On the 2nd ED, the step serial paraffin sections showed that the primitive hindgut appeared as a tube, and the caeca arose as bilateral invaginations in the hindgut mucosa. Then the primitive caeca began to separate from the hindgut resulting in bilateral swelling on both ventrolateral sides of the hindgut wall (Fig. 1A–D). It consisted of unfolded mucosa that composed of pseudostratified columnar epithelium, surrounded by the same mesenchymal tissue layer of the hindgut wall. Many mitotic divisions were observed in both the mucosal epithelium and mesenchymal tissue. The lumen was open and free from any materials (Fig. 1E,F). On the 3^rd^ ED, the caeca appeared as two evaginations from the hindgut. Then the caeca began to elongate creating two tubes. The caecal epithelium was pseudostratified in type, surrounded by undifferentiated mesenchymal tissue and covered with the peritoneal epithelium (Fig. 1G–I).

**Figure 2:**
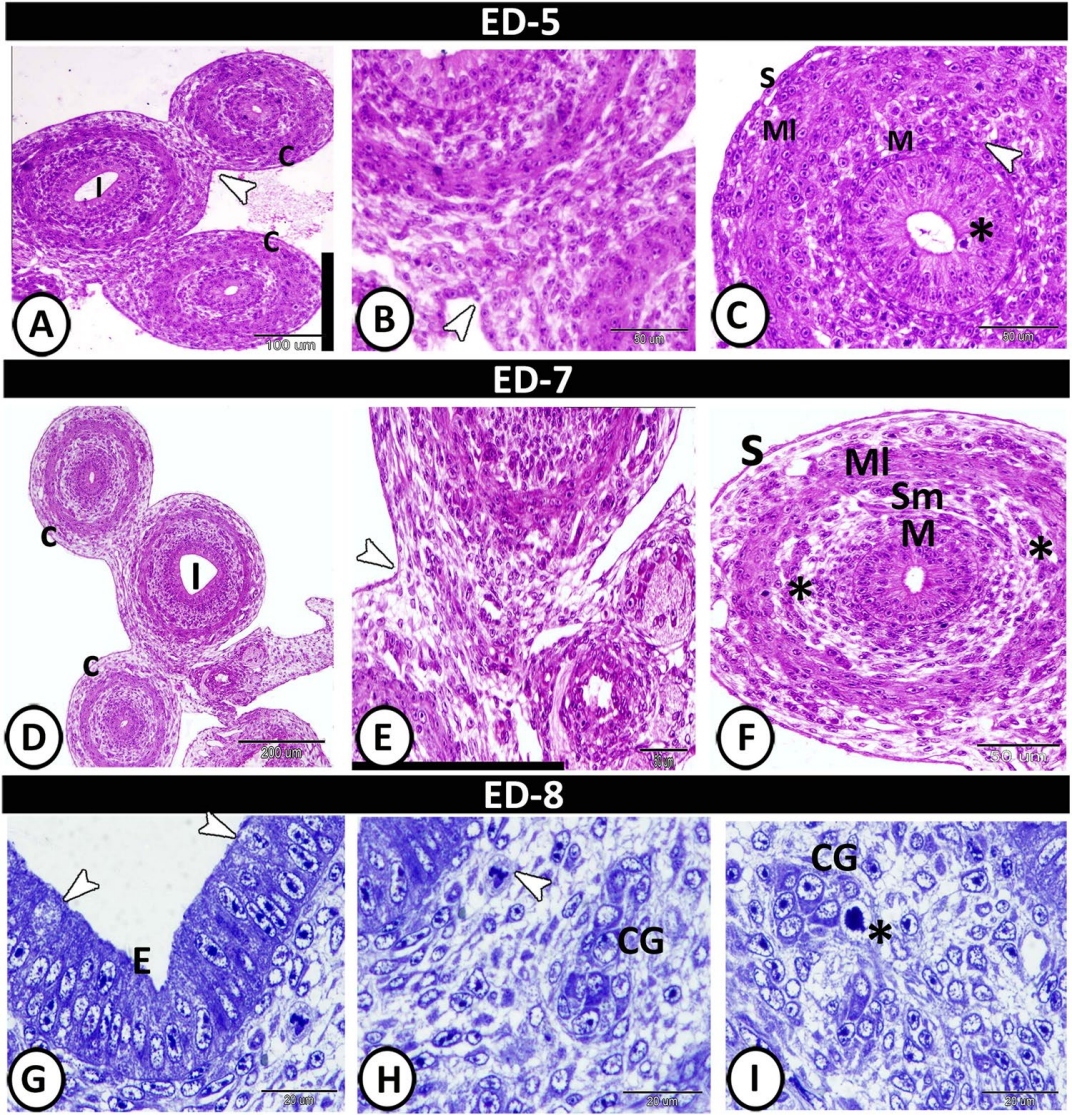
T.S in the caecum on the 5^th^, 7^th^, 8^th^ ED, (A-F) stained by H&E, (G-I) stained by toluidine blue. (**A**,**B**) Partial separation of the serosal layer (arrowheads) of caeca (**C**) and ileum (I). (**C**) The epithelium of caecal mucosa (M) showing mitotic divisions (asterisk), followed by lamina propria/submucosa (arrowhead), muscle layer (Ml) and serosa (S). (**D**) The ileum (I) between the two caeca (**C**). (**E**) Higher magnification showed the incomplete separation of serosa (arrowhead). (**F**) The caecal wall consisted of mucosa (M), submucosa (sm) with the primordia of the caecal glands (*), the muscle layer (Ml) and serosa (S). (**G**) Semithin section showed mucosal epithelium (**E**) with rodlet cells (arrowheads). (**H**,**I**) Submucosa contained macrophage (arrowhead) and primordia of caecal glands (CG) with mitotic divisions (asterisk).

On the 5^th^ ED, the caecal layers were represented by unfolded mucosa, consisted of pseudostratified columnar epithelium with short brush borders and showed many mitotic divisions. The lamina propria/submucosa consisted of undifferentiated mesenchymal tissue. An undeveloped smooth muscle layer with mitotic divisions was observed for the first time at this age and surrounded by a serosa (Fig. 2A–C). On the 7^th^ ED, the lamina propria/submucosa contained the primordia of glands. The muscle layer was more developed, increased in thickness and consisted of outer longitudinal and inner circular layers infiltrated by blood vessels. The serosa was well-developed (Fig. 2D–F). On the 8^th^ ED, the semithin sections showed a weak metachromatic reaction in the apical part of the epithelium. The rodlet cells could be observed amongst the epithelial cells and characterised by their intracytoplasmic inclusions (Fig. 2G). The caecal gland appeared as a small mass of aggregated undifferentiated cells. Many mitotic divisions were observed in the lamina propria/submucosa. The lamina propria was characterised by high vascularity and rich cellular composition (Fig. 2H,I).

**Figure 3:**
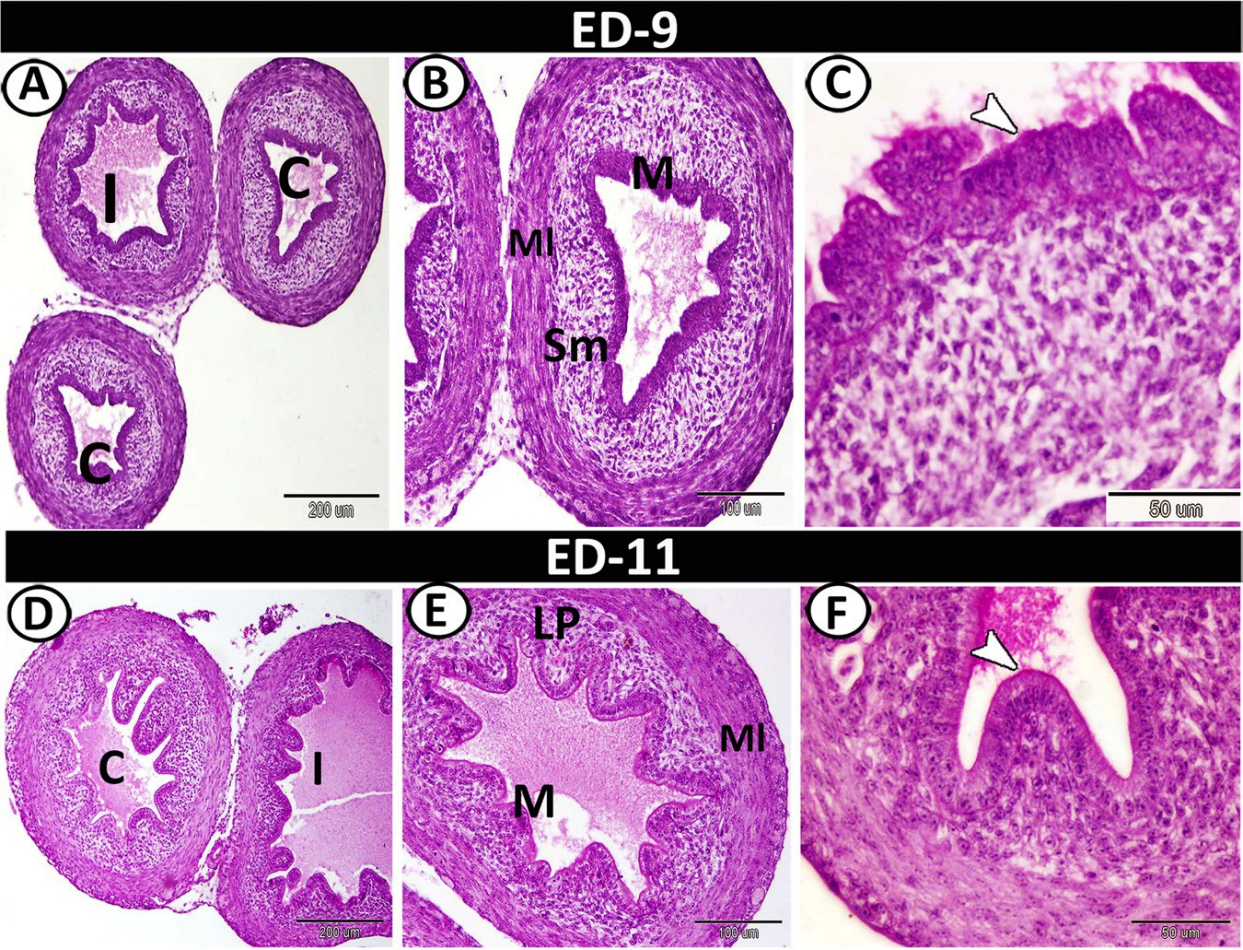
T.S in the proximal part of caeca on the 9^th^ and 11^th^ ED. (**A**,**B**) The beginning of the proximal part of caeca (**C**), the ileum (I) and the caecal layers: mucosa (M), submucosa (sm), and muscle layer (Ml) stained by HE. (**C**) PAS-positive epithelial microvilli (arrowhead). (**D**) The proximal part of the caecum (**C**) and the ileum (**I**). (**E**) The proximal part layers: mucosa (M), lamina propria (Lp) and muscle layer (Ml). (**F**) The proximal part showed PAS-positive brush borders (arrowhead).

On the 9^th^ ED, the caeca could be divided into three parts with more developed layers. The mucosa of the proximal part was made up of 5 to 7 folds per cross-section that were covered by pseudostratified columnar epithelium. The lamina propria/submucosa was formed of undifferentiated mesenchymal tissue. The muscle layer was differentiated into thick inner circular and thin outer longitudinal layers (Fig. 3A,B). The proximal part showed a positive reaction to PAS in its epithelial brush borders (Fig. 3C). On the 11^th^ ED, the mucosal folds of the proximal part increased to 10 folds. The mucosal epithelium was simple columnar in type and showed mitotic divisions (Fig. 3D,E). The lumen was increased in size and was filled with PAS-positive secretions (Fig. 3F).

**Figure 4:**
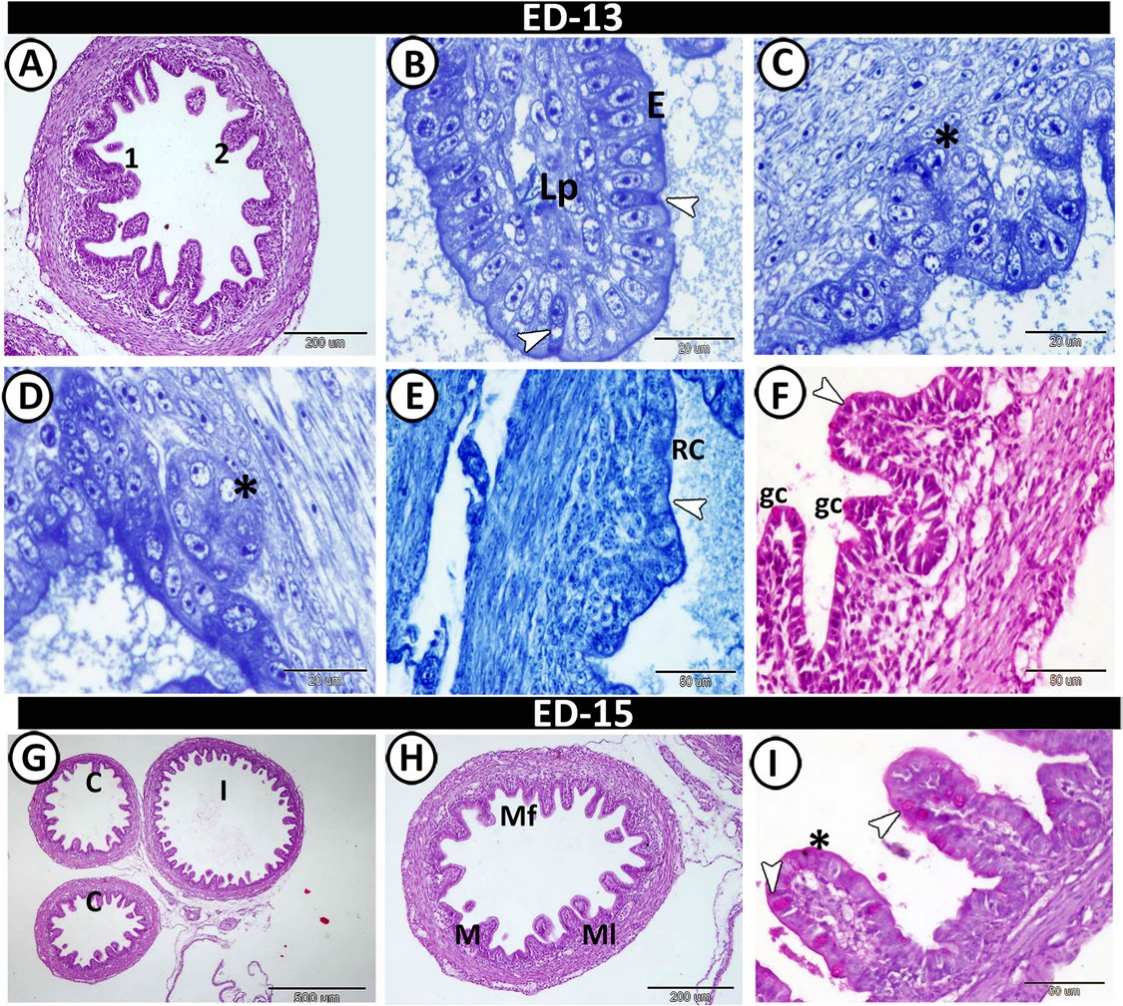
T.S in the proximal part of caeca on the 13^th^ and 15^th^ ED. (**A**) Two shapes of mucosal villi stained by HE; branched finger-shaped villi (1), finger-shaped villi (2). (**B**) Semithin section stained by toluidine blue showed a part of villi occupied with lamina propria (Lp) and covered by simple epithelium (**E**) and rodlet cells (arrowheads). (**C**,**D**) Submucosa with crypts of Lieberkuhn (asterisks). (**E**) Bromophenol blue-positive rodlet cell (RC) and brush borders (arrowhead). (**F**) PAS-positive goblet cell (gc) and brush borders (arrowhead). (**G**) General view of the proximal part of caeca (**C**) and ileum (**I**) stained by HE. (**H**) The wall of the proximal part showed mucosal folds (Mf), mucosa (**M**) and muscle layer (Ml). (**I**) The mucosal folds showed PAS-positive goblet cells (arrowheads) and microvilli (asterisk).

On the 13^th^ ED, the caeca appeared morphologically to be as mature as the adult. A gradual increase in the length of mucosal folds was noticed. In addition, the folds increased in number to 12 folds per cross-section that were variable in shapes, ranged from finger to branched folds. The muscle layer was well-developed, increased in thickness and the outer longitudinal muscle layer was infiltrated by blood vessels (Fig. 4A). The folds were covered by columnar epithelium with goblet cells and were occupied by dense connective tissue lamina propria (Fig. 4B). Semithin sections revealed the presence of crypts of Lieberkuhn that consisted of low columnar epithelium. In addition, rodlet cells were observed between the surface epithelium (Fig. 4B–D). The rodlet cells and the most apical portion of the epithelium exhibited a high affinity for bromophenol blue (Fig. 4E). The few goblet cells and the brush borders of the epithelium showed a strong affinity for PAS (Fig. 4F). On the 15^th^ ED, the mucosal folds increased in number to reach 15 folds per cross-section (Fig. 4G,H). Goblet cells increased in number and showed a strong affinity for PAS (Fig. 4I).

**Figure 5:**
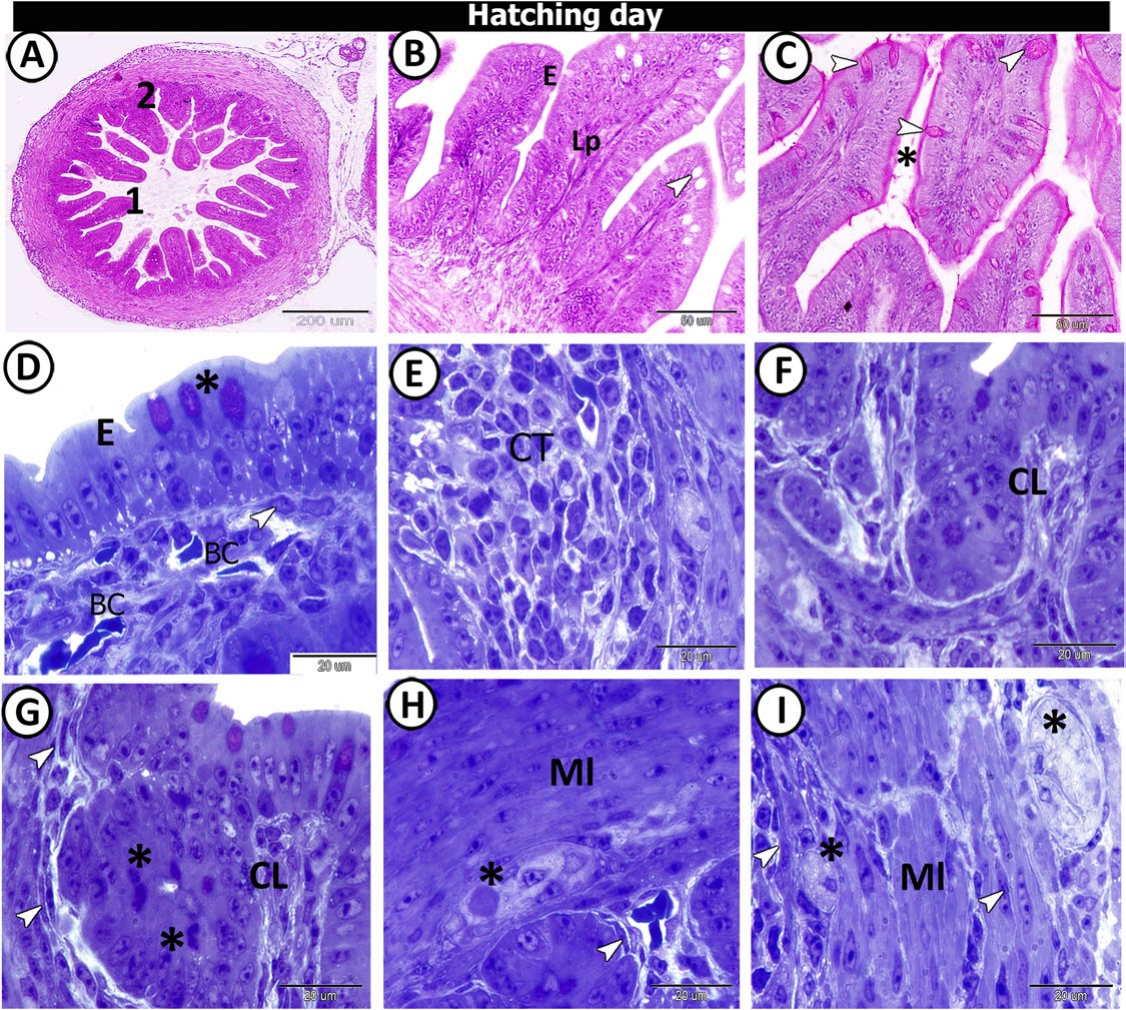
T.S in the proximal part of caeca at hatching day. (**A**) Two shapes of villi (1&2). (**B**) The finger-shaped villi filled with lamina propria (Lp) and covered by epithelium (**E**) with goblet cell (arrowhead). (**C**) PAS-positive goblet cells (arrowheads) and brush borders (asterisk). (**D**) Semithin section stained by toluidine blue (TB) showed the surface epithelium (E) dispersed by goblet cells (asterisk). Note, telocytes (arrowhead) in association with blood capillaries (BC). (**E**,**F**) Lamina propria showed crypts of Lieberkuhn (CL), and caecal tonsils (CT). (**G**) Telocytes (arrowheads) surrounded crypts of Lieberkuhn (CL) with endocrine cells (asterisks). (**H**,**I**) Muscle layer (Ml) infiltrated by nerve bundles (asterisks) and telocytes (arrowheads).

On the hatching day, two forms of villi could be observed: (1) finger-shaped villi and (2) branched finger-shaped ones. The number of goblet cells increased compared with the samples taken at the previous developmental ages and the number of villi also increased to reach 14 villi per cross-section (Fig. 5A,B). The goblet cells and brush borders showed a strong affinity for PAS. Furthermore, the semithin sections showed that the surface epithelium was simple columnar in type, dispersed with metachromatic goblet cells (Fig. 5D). Typical caecal tonsils (CTs) could be identified in this age in the form of massive aggregations of lymphocytes (Fig. 5E). The connective tissue core of the mucosal villi contained strands of smooth muscle fibres, telocytes, blood capillaries, and lymphocytes (Fig. 5D,G). Notably, the crypts of Lieberkuhn were well-defined and were formed of low simple columnar epithelium, goblet cells, and enteroendocrine cells (Fig. 5F,G). Large nerve bundles and blood vessels were observed in the tunica muscularis (Fig. 5H,I).

**Figure 6:**
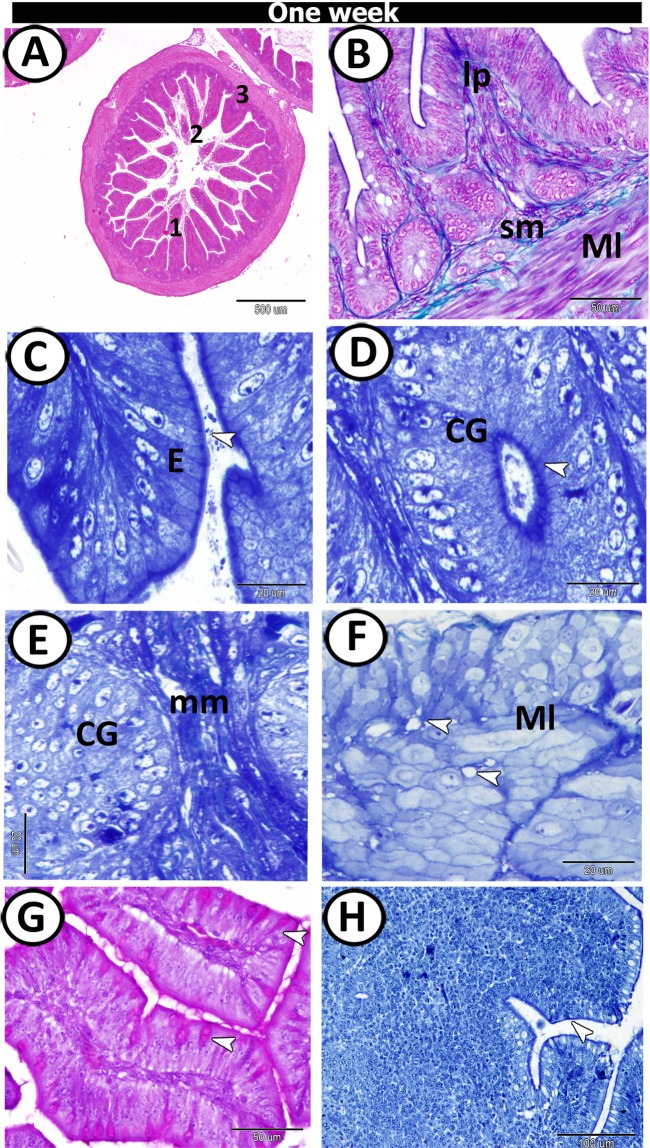
T.S in the proximal part of caeca at one-week post-hatching. (**A**) Shapes of mucosal folds; finger 1, leaf-like 2 & tongue shape 3. (**B**) The wall of the caecum stained by Crossmon’s Trichrome showing muscle layer (Ml), submucosa (sm) and lamina propria (Lp). (**C**-**E**) Semithin section showed the epithelium (**E**) of the mucosal fold and caecal glands (CG) with prominent microvilli (arrowhead), surrounded by muscularis mucosa (mm). (**F**) Outer and inner muscle layers (Ml) separated by fat cells (arrowheads). (**G**) PAS-positive goblet cells (arrowheads). (**H**) Bromophenol blue-positive apical epithelial surface (arrowhead).

At one week post-hatching, the proximal part of the caecum exhibited the same embryonic layers, but with more advanced development. The lumen was filled with villi. The villi were variable in shape: (1) tongue-shaped, (2) long finger-shaped and (3) leaf-like villi (Fig. 6A). The collagenous connective tissue was distributed mainly in the lamina propria/submucosa and between the muscle layers (Fig. 6B). The villi were covered by simple columnar cells with well-distinct brush borders (Fig. 6C). The crypts of Lieberkuhn were composed of simple columnar epithelium with brush borders. Bundles of muscularis mucosa were observed between the crypts (Fig. 6D,E). Semithin sections also showed a well-developed muscle layer; consisted of a thick inner circular layer separated by fat cells and very thin outer longitudinal muscle fibres infiltrated by branches of blood vessels (Fig. 6F). Numerous goblet cells were scattered at the surface epithelium and exhibited a positive reaction to PAS (Fig. 6G). Moreover, rodlet cells and the apical parts of the epithelium showed a positive reaction to bromophenol blue (Fig. 6H).

**Figure 7:**
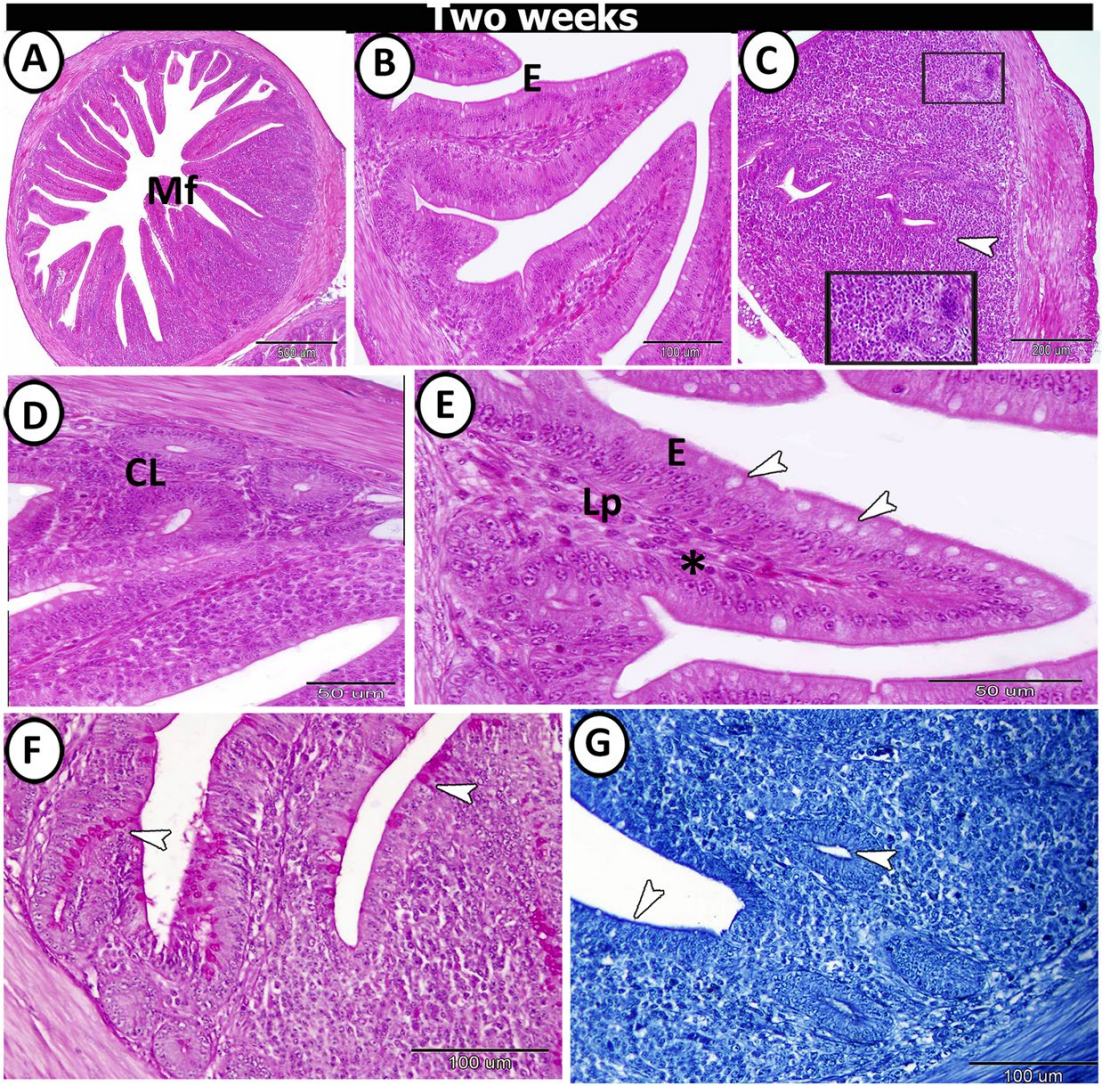
T.S in the proximal part of caeca at two weeks post-hatching. (**A**,**B**) Many finger-shaped villi (MF) covered by simple epithelium (**E**). (**C**,**D**) The lamina propria contained crypts of Lieberkuhn (CL) and caecal tonsils (CT) (arrowhead, boxed areas). (**E**) The villi filled with lamina propria (Lp) and covered by epithelium (**E**) infiltrated by goblet cells (arrowheads) and intraepithelial lymphocytes (IELs) (asterisk). (**F**) PAS-positive goblet cells (arrowheads). (**G**) Bromophenol blue-positive brush borders and apical portion of the crypts of Lieberkuhn (arrowheads).

At two weeks post-hatching, the villi of the proximal part exhibited two forms: (1) tongue-shaped villi containing tonsils and (2) finger-shaped ones without tonsils (Fig. 7A,B). The caecal tonsils (CTs) appeared as a massive aggregation of lymphocytes (Fig. 7C). The crypts of Lieberkuhn were well-developed and consisted of low simple columnar cells with few goblet cells (Fig. 7D). The surface epithelium lining the villi consisted of columnar cells with microvilli, dispersed with many goblet cells and showed a high number of intra-epithelial lymphocytes (Fig. 7E). The apical cytoplasm of both surface epithelium and epithelium of crypts showed a positive reactivity towards bromophenol blue and PAS. Furthermore, the goblet cells also showed a strong affinity for PAS (Fig. 7F,G).

### Immunohistochemical analysis

**Figure 8:**
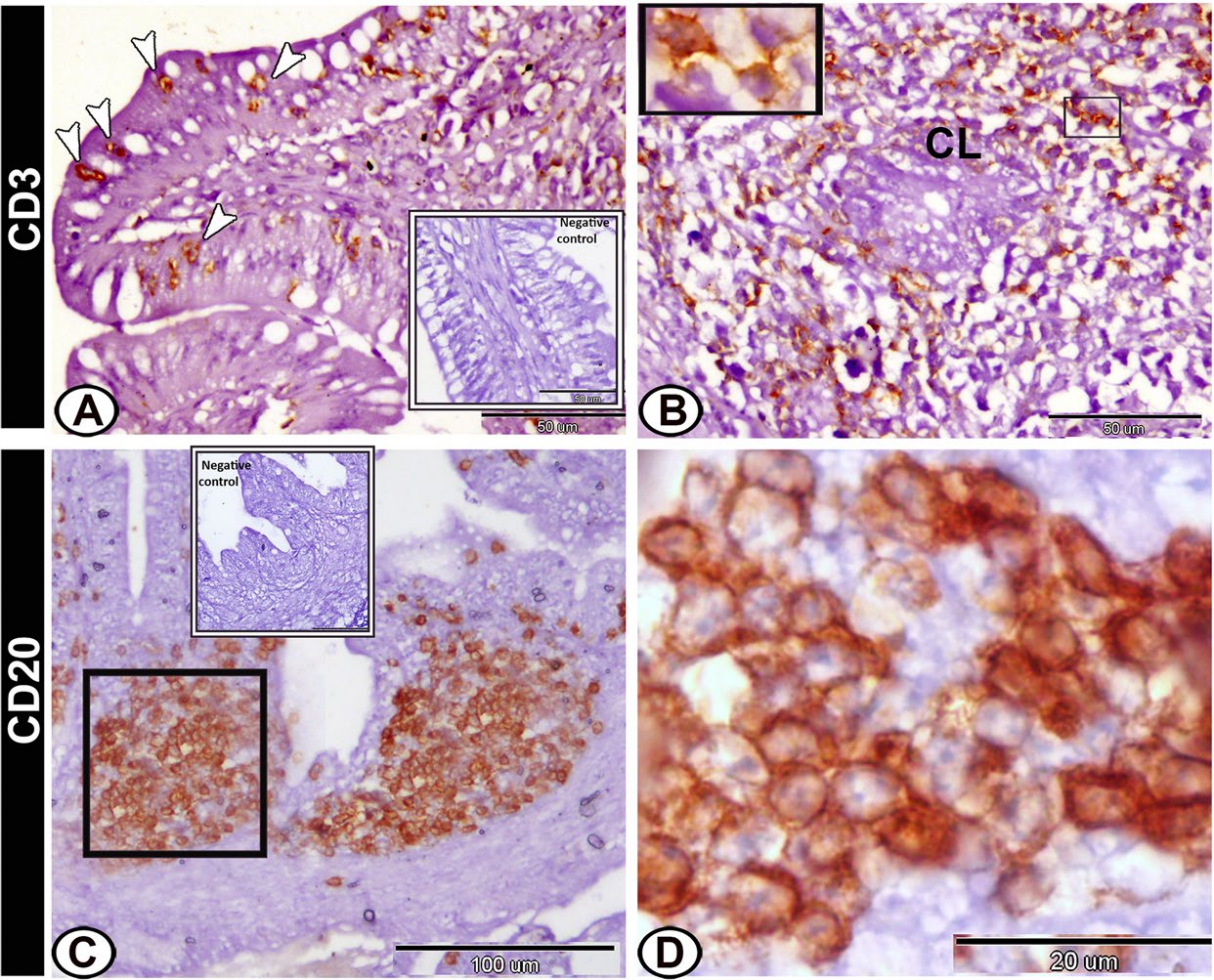
Immunohistochemical expression pattern of CD3 and CD20 in the proximal part of caeca at hatching day. (**A**) Intraepithelial lymphocytes (arrowheads) expressed immunopostivity to CD3. (**B**) Lamina propria showed CD3-immunopositive T-lymphocytes (boxed areas) around the crypts of Lieberkuhn (CL). (**C**,**D**) CD20-positive B-lymphocytes were immunolocalised in the caecal tonsils (boxed area). Note, the negative control (boxed area) in **A**,**C**.

**Figure 9:**
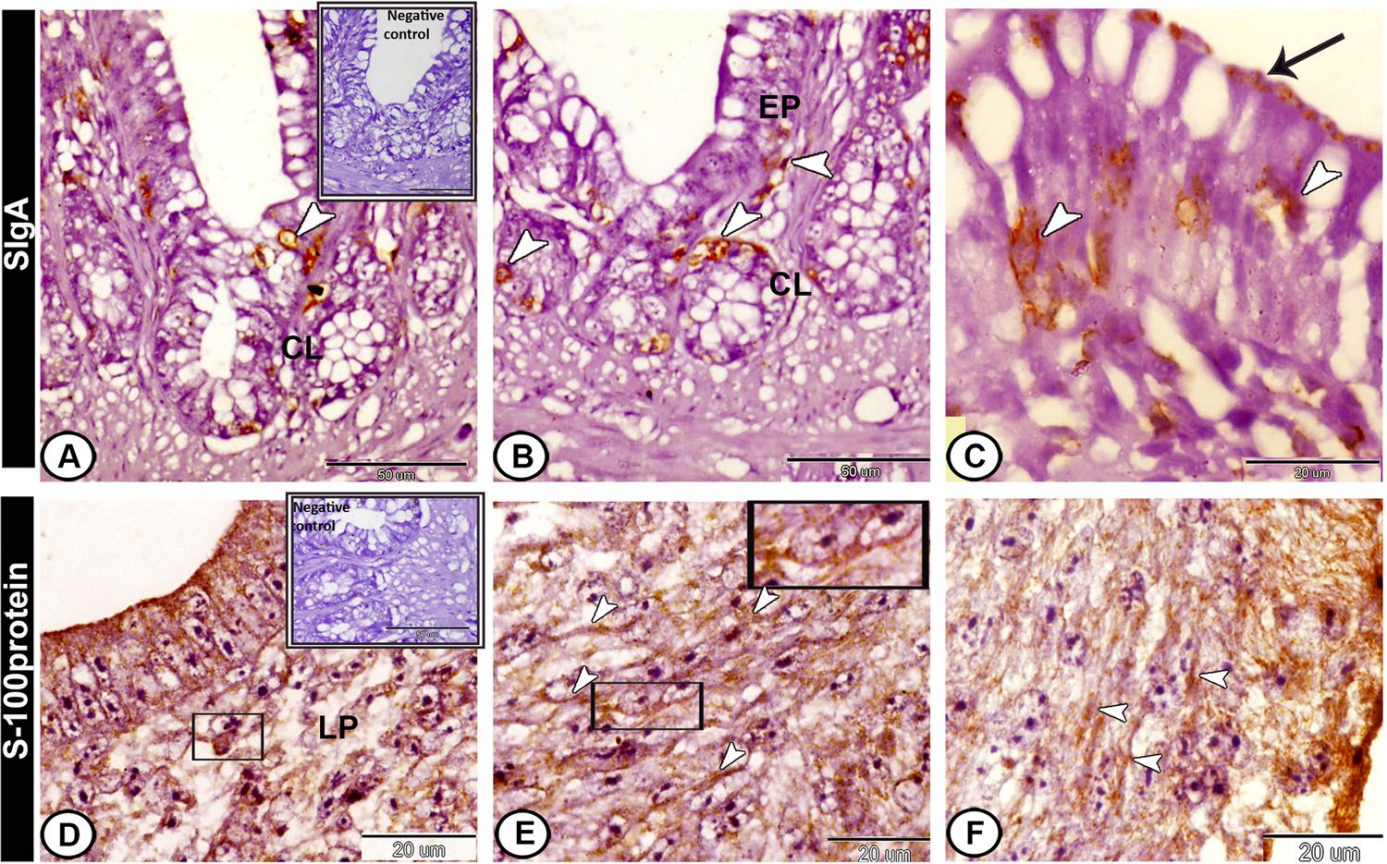
Immunohistochemical expression pattern of Secretory immunoglobulin A (SIgA) and s100 protein in the proximal part of caeca at hatching day. (**A**,**B**) SIgA-positive cells (arrowheads) in the lamina propria (LP) under the epithelium (EP) and the crypts of Lieberkuhn (CL). (**C**) Positive-SIgA in the epithelial cells (arrowheads). Note, an intense staining reaction in the outer epithelial membrane (arrow). (**D**) S-100 protein-immunopositive dendritic cells (boxed area) in the lamina propria (LP). (**E**,**F**) S-100 protein-positive telocytes (arrowheads, boxed areas) between the muscle layers. Note, the negative control (boxed area) in A,D.

On the hatching day, CD3-immunopositive T-lymphocytes were highly concentrated in the lamina propria. In addition, intraepithelial T-lymphocytes expressed immunopostivity to CD3 (Fig. 8A,B). However, CD20-positive B-lymphocytes were immunolocalised mainly in the caecal tonsils (Fig. 8C,D). Secretory IgA-positive cells were observed mostly in the epithelial cells, lamina propria and the crypts of Lieberkuhn. An intense staining reaction was also seen in the outer epithelial membrane (Fig. 9A–C). The dendritic cells in the lamina propria expressed positive immunoreaction to S-100 protein (Fig. 9D). Moreover, S-100 protein-positive telocytes were demonstrated between the muscle layers (Fig. 9E,F).

### Scanning electron microscopy

**Figure 10:**
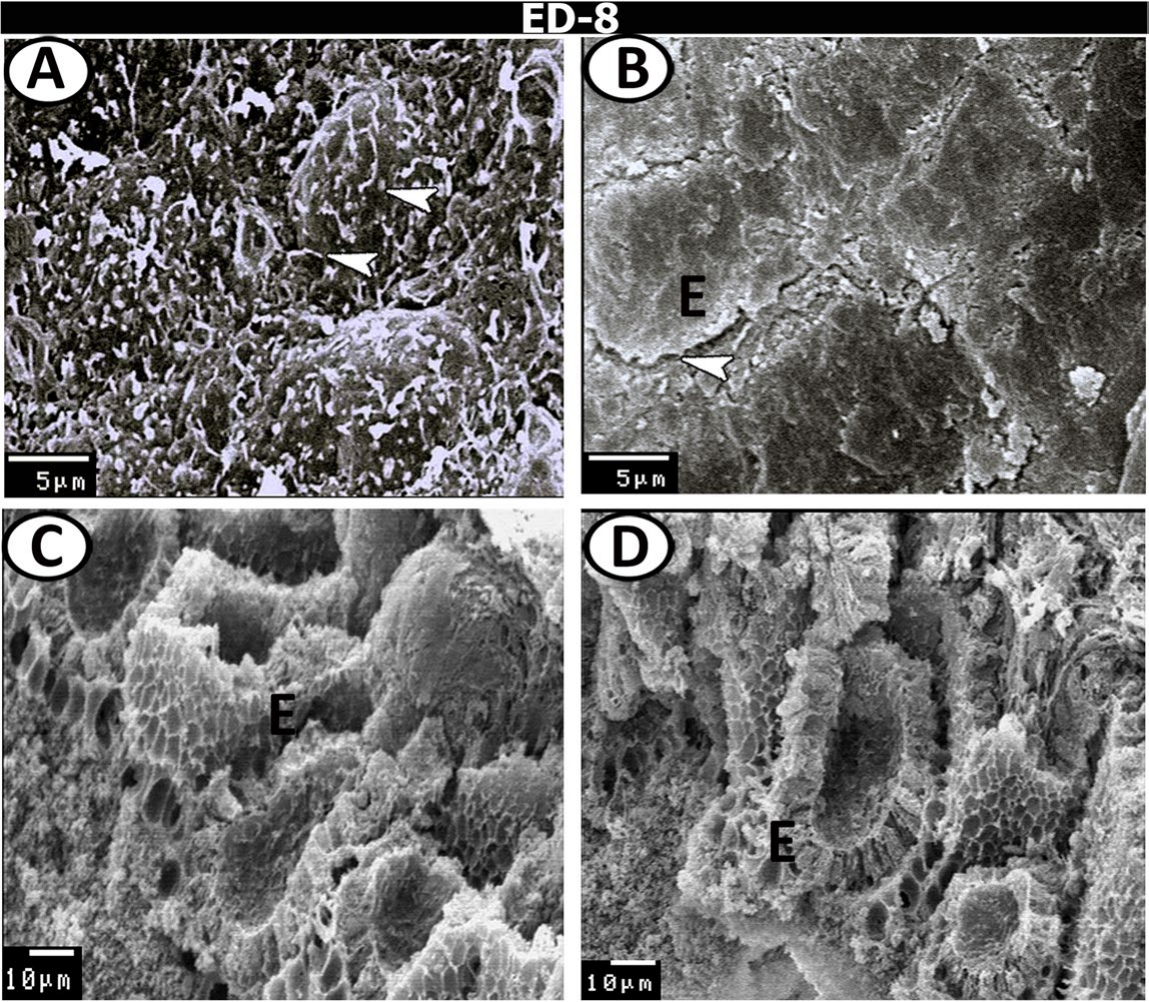
Scanning electron microscopy (SEM) of the caeca on the 8^th^ ED. (**A**) The surface epithelium covered by individual cilium (arrowheads). (**B**) Surface view showing flattened polyhedral epithelium (**E**) with serrated edges (arrowhead). (**C**,**D**) Lateral view showing the epithelium (**E**) and honeycomb-shaped villi.

**Figure 11:**
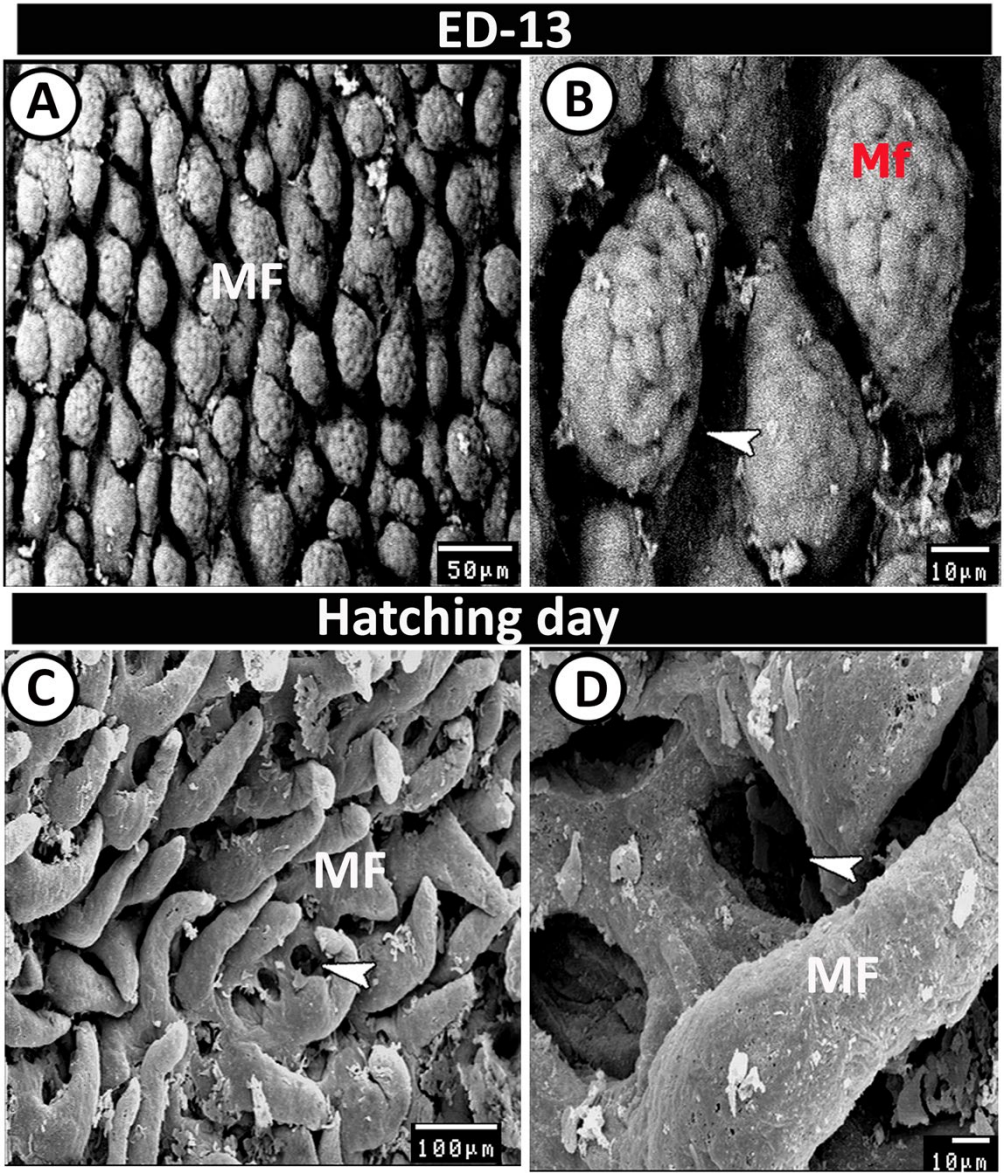
SEM of the proximal part of caeca on the 13^th^ ED and day of hatching. (**A**,**B**) Mucosal folds (Mf) with honeycomb villus surface. Note deep cavities of crypts of Lieberkuhn (arrowhead). (**C**,**D**) Many mucosal folds (MF) with pores for crypts of Lieberkuhn (arrowheads).

**Figure 12:**
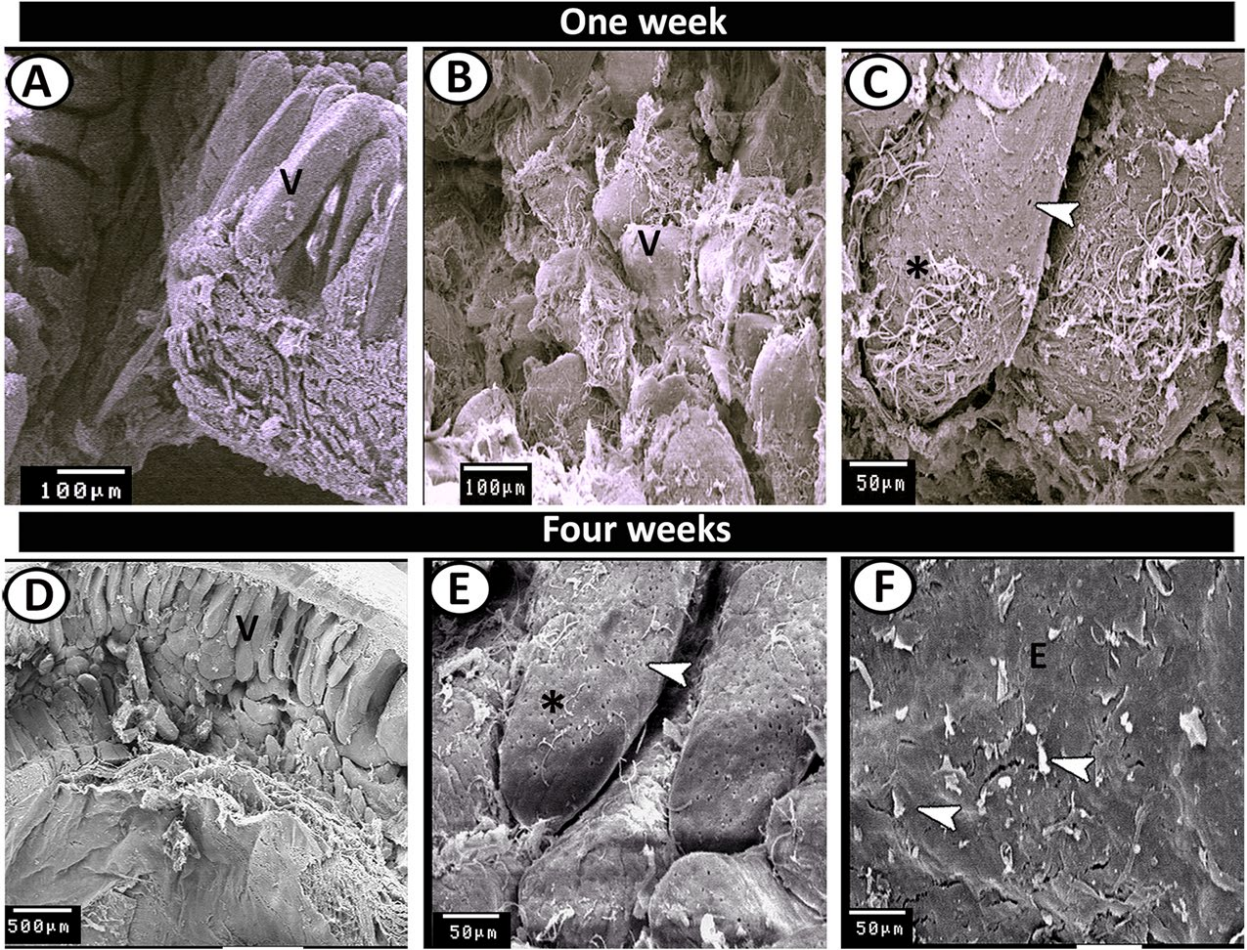
SEM of the proximal part of caeca at one and two weeks post-hatching. (**A**–**C**) The caecal villi (V) covered by cilia (asterisk). Note, pores of goblet cells (arrowhead). (**D**,**E**) A high number of the villi (V) in two-weeks quail showed cilia (asterisk) and numerous pores of goblet cells (arrowhead). (**F**) The surface epithelium (**E**) covered by individual cilium (arrowheads).

On the 8^th^ ED, the villi were few, short, and slightly flattened. Individual cilia covering the epithelium were observed (Fig. 10A). The surfaces of the villi exhibited a corrugated appearance (Fig. 10B). The lateral view of the mucosal surface showed a honeycomb-like appearance along the caecal tube (Fig. 10C,D). On the 13^th^ ED, the mucosal surface was formed of many dome-shaped villi, separated by deep cavities. Higher magnification of the surface epithelial cells showed the presence of numerous short microvilli and few pores for the goblet cells (Fig. 11A,B). On the hatching day, the mucosal surface showed numerous and long finger-shaped villi, with many pores for goblet cells. Crypts of Lieberkuhn orifices were found so deeply between the villi (Fig. 11C,D). At one week post-hatching, more development was observed in the pattern of organisation of villi, which was associated with an increase in their number and length. Moreover, many goblet cell pores and numerous long cilia were scattered on the mucosal surface (Fig. 12A–C). At four weeks post-hatching, the proximal part showed condensed finger-shaped villi with numerous goblet cell pores and individual cilia (Fig. 12D–F).

### Transmission electron microscopy

**Figure 13:**
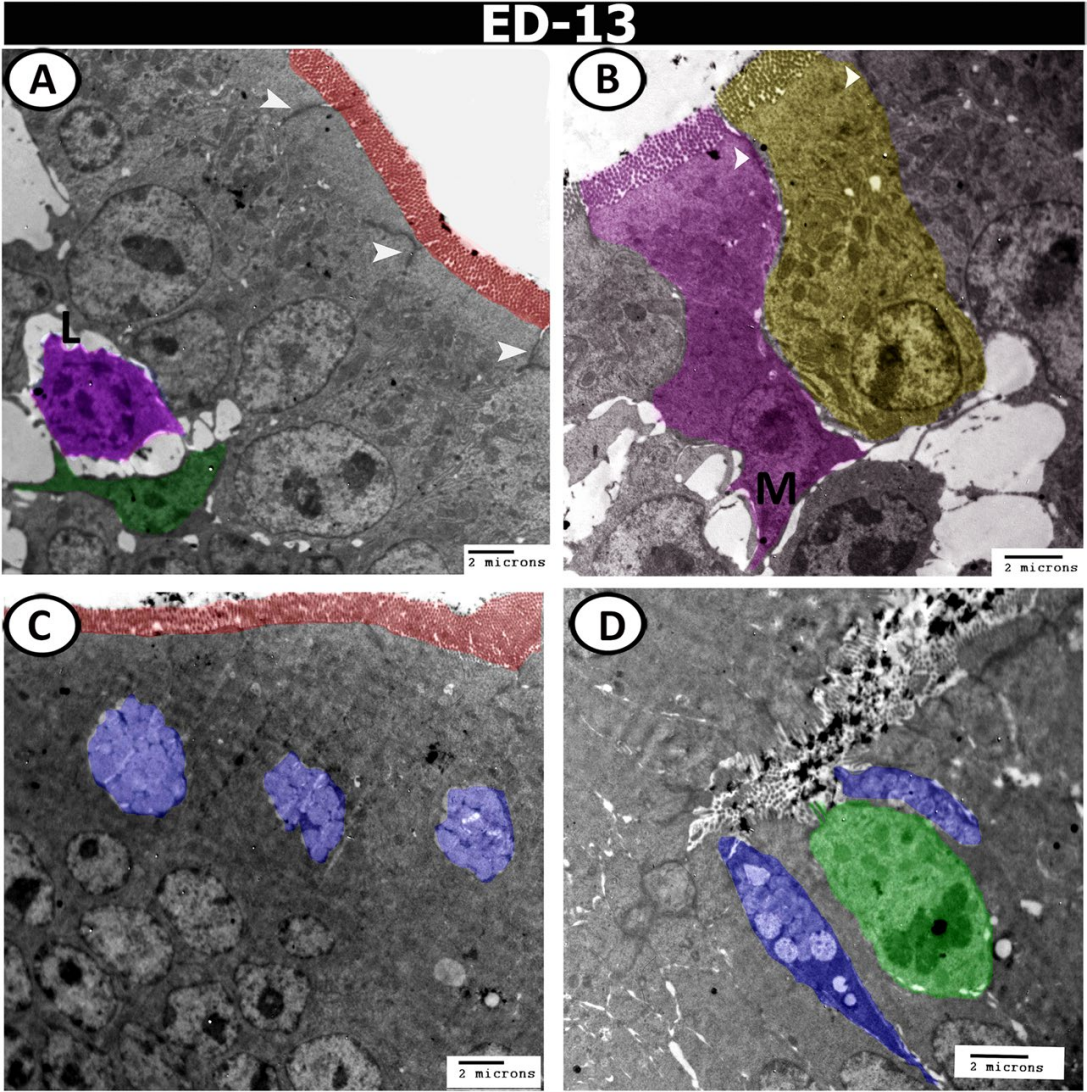
Digital coloured transmission electron microscopic (TEM) images of the proximal part of caeca on the 13^th^ ED. (**A**,**B**) The epithelium consisted of simple columnar cells (yellow colour) with brush borders (red colour), and connected to the neighbouring cells by desmosomes (arrowheads). Note M-cell (M, pink colour), and the infiltration of the epithelium by dendritic cell (green) and lymphocytes (L, violet). (**C**,**D**) Goblet cells (blue colour), and rodlet cell (green colour) are distributed in the epithelium.

**Figure 14:**
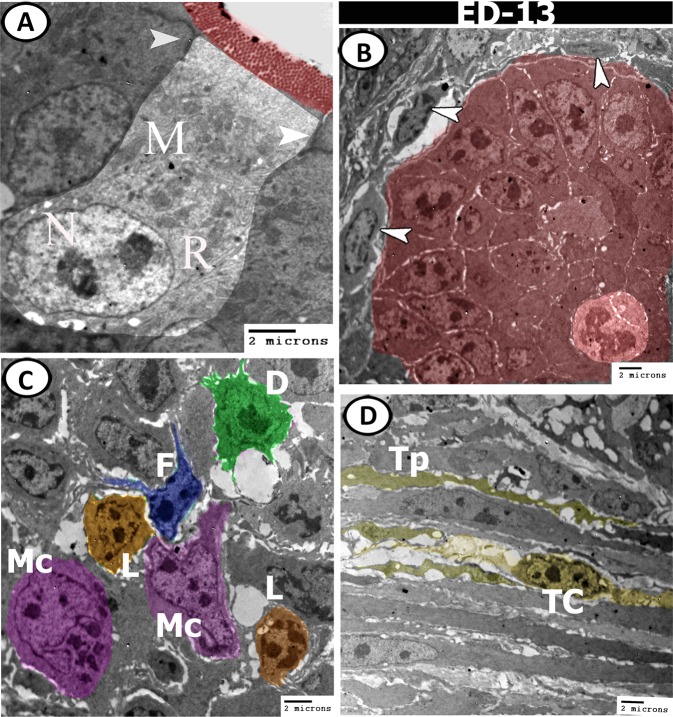
Digital coloured TEM images of caecum on the 13^th^ ED. (**A**) Surface epithelium with euchromatic nucleus (N), contained rough endoplasmic reticulum (R) and mitochondria (M) and connected by desmosomes (arrowheads). (**B**) The crypts of Lieberkuhn (red colour) are surrounded by smooth muscle cells (arrowheads). (**C**) Submucosa contained macrophages (pink, Mc), lymphocytes (orange, L), dendritic cell (green, D), and fibroblast (blue, F). (**D**) Muscle layer infiltrated by telocytes (TC) and their telopodes (Tp) (yellow colour).

On the 13^th^ ED, the simple columnar epithelium possessed oval euchromatic nuclei with 1–2 distinct nucleoli and covered with microvilli. These cells were connected by desmosomes. Numerous lymphoid cells such as intraepithelial lymphocyte (IEL) and dendritic cells were identified at the basal border of the epithelium (Fig. 13A). Immature M- cells were covered by microvilli and attached with the neighbouring epithelial cells by desmosomes. The M-cells possessed numerous mitochondria and euchromatic nucleus with distinct nucleolus (Fig. 13B). The epithelium was infiltrated by many goblet cells that characterised by the presence of many mucous globules. A rodlet cell was also seen amongst the epithelium that contained a thick capsule and many inclusions (rodlets) (Fig. 13C,D). Numerous mitochondria were concentrated at the apical portion of the epithelial cells, in addition to the presence of many profiles of rER around the nucleus (Fig. 14A). Crypts of Lieberkuhn were consisted of low columnar epithelium with oval euchromatic nuclei and distinct nucleoli and were surrounded by smooth muscle cells (Fig. 14B). At this age, atypical caecal tonsils (CTs) appeared in the form of lymphoid aggregation (encapsulated form). This lymphoid aggregation consisted of macrophages, lymphocytes, and dendritic cells. The macrophages possessed large eccentric euchromatic nuclei and intracytoplasmic phagocytosed materials. The lymphocytes were characterised by a large nucleus with a patchy chromatin pattern and a thin rim of cytoplasm. The dendritic cells resembled the lymphocytes but with many short processes (Fig. 14C). Telocytes were found between the muscle fibres and consisted of a spindle-shaped body with euchromatic nucleus and two long processes called telopodes (Fig. 14D).

**Figure 15:**
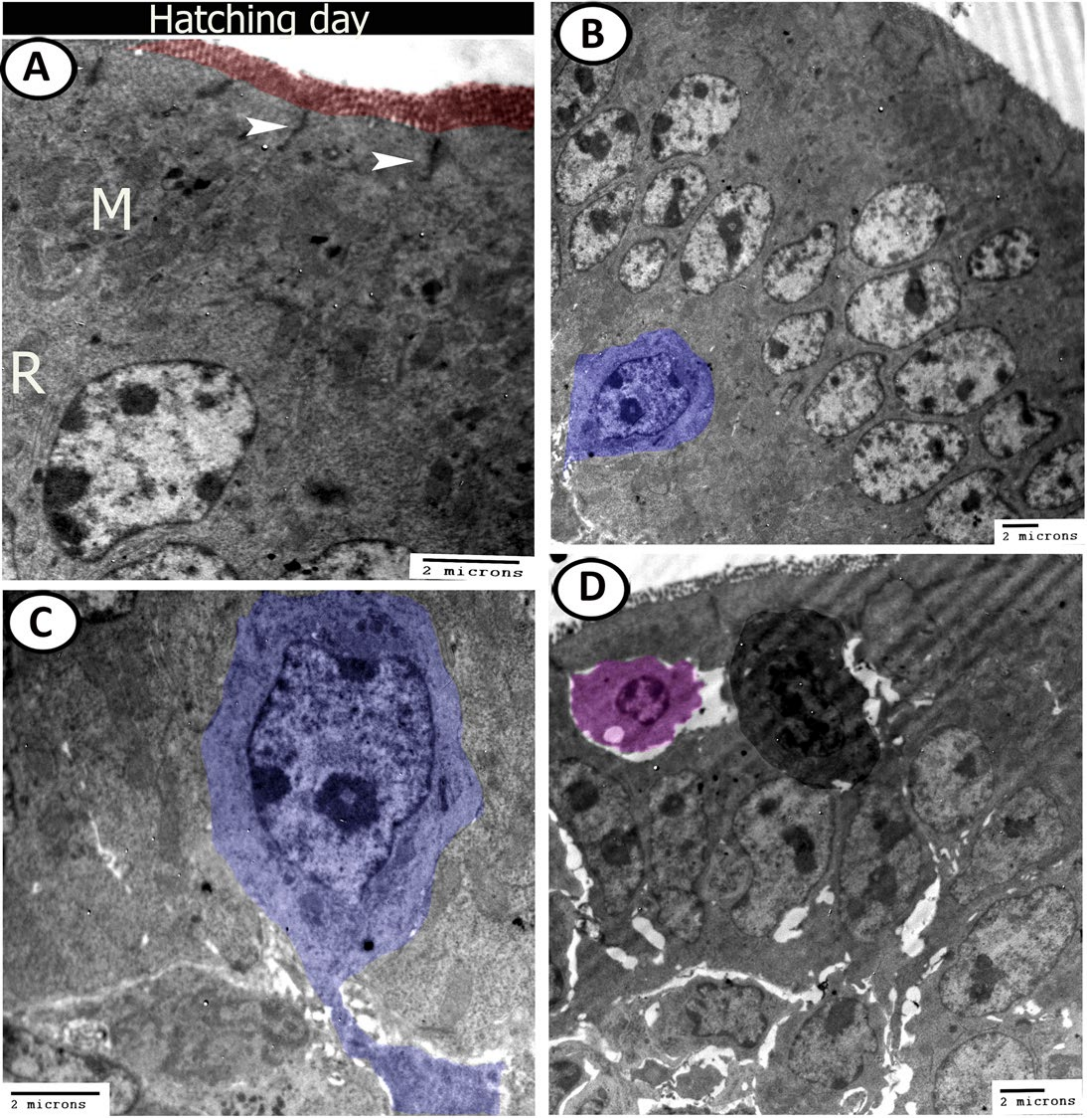
Digital coloured TEM images of caecum at hatching day. (**A**) The epithelium connected by desmosomes (arrowheads), covered by microvilli (red), and the cytoplasm contained rough endoplasmic reticulum (R) and mitochondria (M). (**B**,**C**) Surface epithelium with neuroendocrine cell (blue colour). (**D**) Intraepithelial lymphocyte (pink) and basophile leukocyte (dark colour) are migrated to the epithelium.

**Figure 16:**
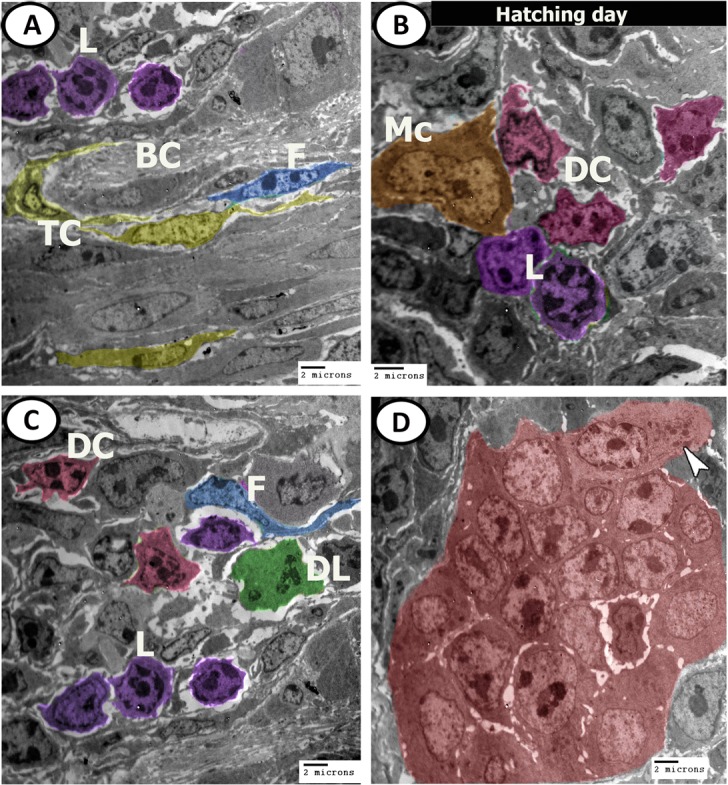
Digital coloured TEM images of caecum at hatching day. (**A**–**C**) Submucosa contained clusters of lymphocytes (violet, L), dendritic cell (pink, DC), Macrophages (orange, Mc), developing leukocytes (green, DL) and blood capillaries (BC) surrounded by telocytes (yellow, TC) and fibroblast (blue, F). (**D**) Crypts of Lieberkuhn (red) with enteroendocrine cells contained electron-dense granules (arrowhead).

On the day of hatching, the surface epithelium was covered by brush borders and connected by desmosomes. Their cytoplasm contained many mitochondria and profiles of rER (Fig. 15A). A closed type neuroendocrine cell appeared near the basement membrane and extended a process into the submucosa. Its nucleus contained euchromatin with a distinct nucleolus and the cytoplasm contained many mitochondria (Fig. 15B,C). Intraepithelial lymphocytes (IELs) and basophil leukocyte were seen within the epithelial layer (Fig. 15D). Moreover, the submucosa was infiltrated by numerous immune cells including lymphocytes, macrophages, dendritic cells, developing leukocytes and blood capillaries surrounded by telocytes and fibroblasts (Fig. 16A–C). The crypts of Lieberkuhn contained endocrine cells that possessed many mitochondria, rER, and dense granules (Fig. 16D).

**Figure 17:**
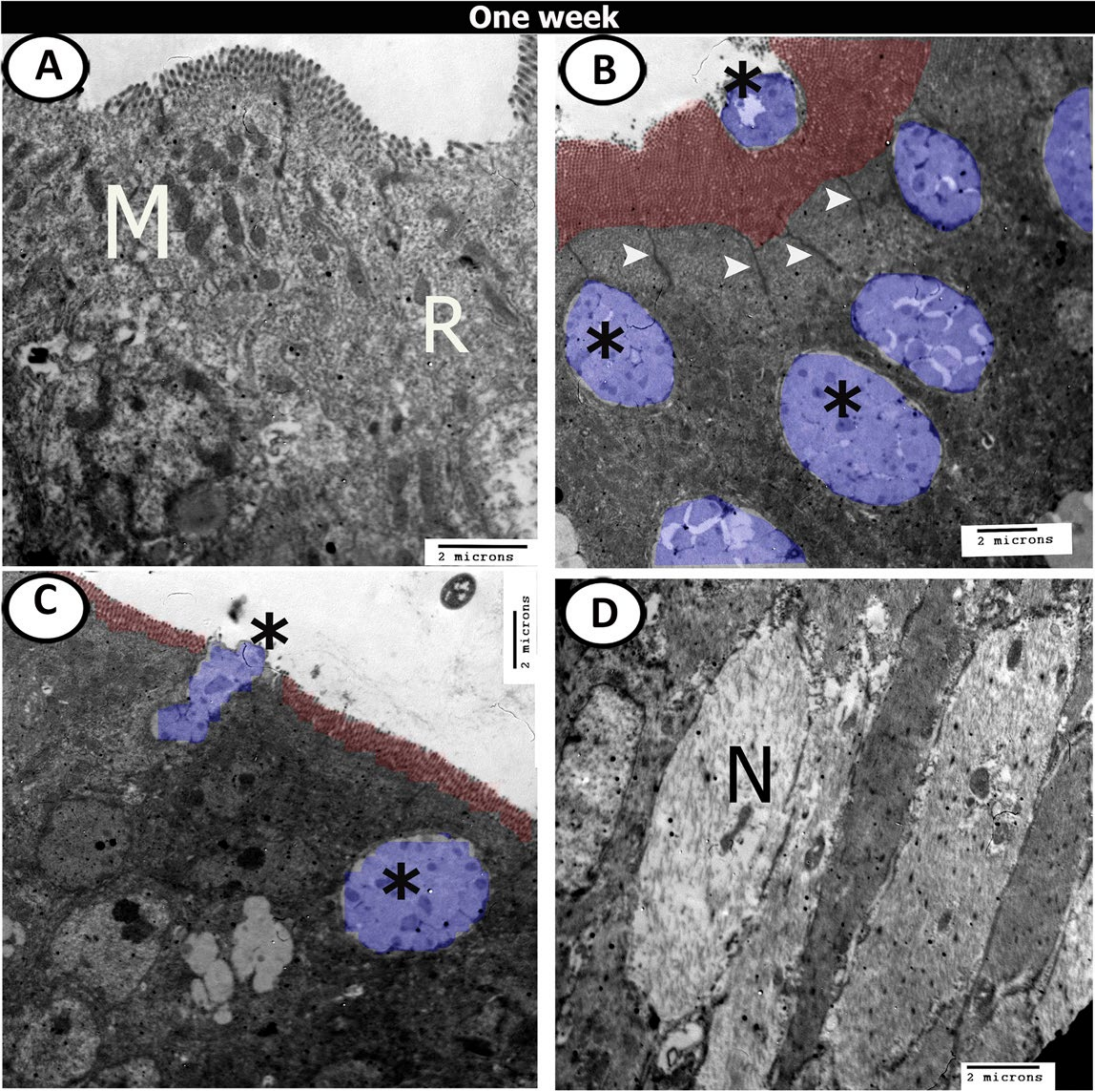
Digital coloured TEM images of caecum at one-week post-hatching. (**A**-**C**) Simple epithelium contained rough endoplasmic reticulum (R), mitochondria (M), dispersed by goblet cells (blue colour, asterisks), connected with desmosome (arrowheads) and covered by microvilli (red colour). (**D**) Bundles of nerve fibres (N) between the muscles.

**Figure 18:**
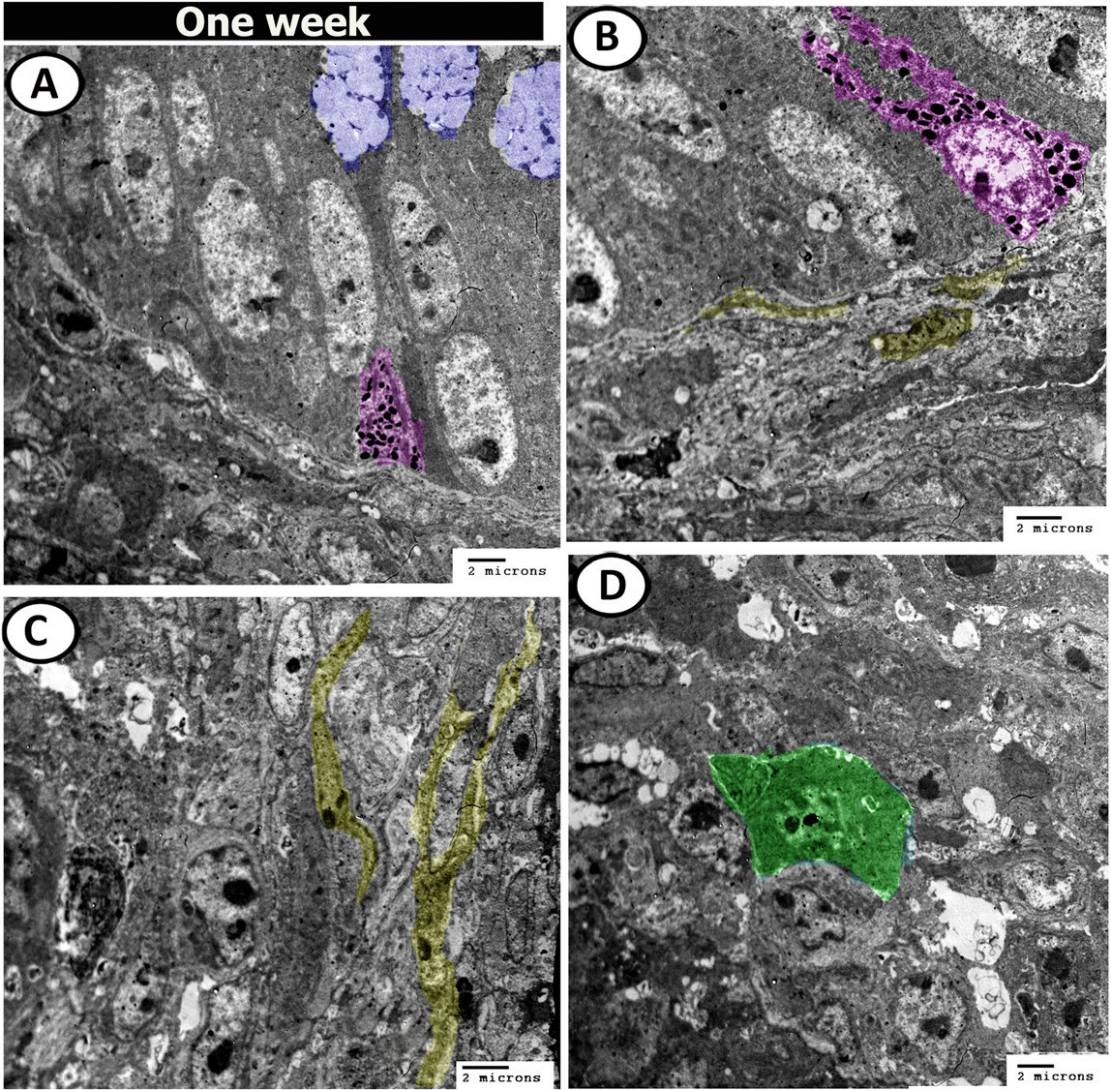
Digital coloured TEM images of caecum at one-week post-hatching. (**A**,**B**) Enteroendocrine cells with dense granules (purple colour), goblet cells (blue colour) and telocytes (yellow colour) under the basement membrane. (**C**,**D**) telocytes (yellow) and macrophage (green) in the submucosa.

At one week post-hatching, the surface epithelial cells were covered by dense microvilli (Fig. 17A). Interestingly, many mature goblet cells containing dense secretory granules were observed at different levels in the epithelial layer and some of them released their secretions into the lumen (Fig. 17B,C). Bundles of nerve fibres were observed in the lamina propria (Fig. 17D). Closed type enteroendocrine cells also appeared in the epithelium near the basement membrane and possessed euchromatic nuclei and many electron-dense intracytoplasmic granules (Fig. 18A,B). The submucosa contained telocytes (Fig. 18B,C), and macrophage (Fig. 18D).

### Morphometric measurements

All the morphometric parameters of the caecum during both pre- and post-hatching periods were expressed in Table (1).

**Table 1:** The morphometric measurements of the caecum during both pre- and post-hatching periods. The values were expressed as mean (M) ± standard error (SE). ED: embryonic day; H.D: hatching day; W: week.

Ages/Parameters (µm)	ED5	ED7	ED9	ED11	ED13	ED15	H.D.	1 W.	2 W.	4 W.
Diameter of caeca	274.429 ± 15.4	257.006 ± 3.9	353.97 ± 19.8	533.67 ± 12.3	641.239 ± 26.7	596.537 ± 40.16	811.9 ± 50.7	1791.42 ± 48.0	1793.356 ± 57.9	1753.57 ± 43.3
Thickness of muscle layer	52.319 ± 8.6	52.73 ± 4.8	37.55 ± 4.2	61.155 ± 9.0	60.97 ± 13.9	70.57 ± 6.7	78.35 ± 12.8	88.24 ± 13.49	119.191 ± 10.25	180.95 ± 11.13
Villi height	------	------	48.019 ± 13.0	70.195 ± 18.8	90.974 ± 40.7	105.45 ± 12.17	180.49 ± 23.8	310.115 ± 20.26	435.367 ± 19.9	436.185 ± 21.66
Epithelium thickness	33.562 ± 3.0	27.30 ± 2.33	17.6822 ± 3.14	17.92 ± 4.8	18.49 ± 2.3	19.68 ± 1.3	22.52 ± 3.9	30.74 ± 7.9	31.78 ± 7.8	35.923 ± 9.1
Lumen diameter	26.992 ± 5.6	34.565 ± 4.44	127.028 ± 13.3	205.8 ± 21.8	368.015 ± 17.9	351.76 ± 34.8	300.18 ± 27.6	643.231 ± 25.9	561.193 ± 14.3	643.892 ± 8.67

## Discussion

The caecum is exposed to the continuous invasion of antigens of extracaecal origin since it receives the back flowing urine from the urodeum of the cloaca through the colon. Thus, immunological surveillance against foreign microorganisms is necessary^16^. The morphological features of caecum had been investigated in many adult species of birds^15,16,23,32,38^. However, few authors have dealt with the embryonic development of the caecum. This study describes in details the development of the histomorphological characteristics of the caecum of the Japanese quail. The primordia of caeca appeared on 2^nd^ ED and by 7^th^ ED, the primordia of glands could be demonstrated. On 9^th^ ED, the caeca began to divide into three parts. With age, the number and height of the villi increased, the lumen was filled with PAS-positive secretion. M-cells, telocytes, crypts of Lieberkuhn, and caecal tonsils (CTs) were observed on the 13^th^ ED. Post-hatching, crypts of Lieberkuhn were well-defined and the lumen was filled with villi. The surface epithelium lining the villi consisted of simple columnar cells with microvilli, dispersed with many goblet cells and showed a high number of intra-epithelial T-lymphocytes and basophils. Moreover, the caecal immune structures of quails were more developed in post-hatching in comparison to those in the pre-hatching period.

The mucosa of the proximal part of the caecum from the 13^th^ ED until hatching was made up of many villi of variable length that facilitate the absorption. Some studies supported this theory and found that the proximal part of the caecum of birds had well-developed villi and brush borders and was able to transport the amino acids and sugars against the concentration gradient by a mechanism similar to those in small intestine^30^. Upon hatching, the villi were crowded and nearly obliterating the lumen. This pattern could be involved in the filtering mechanism during the filling of the caecum as found in domestic chicken^31^ and geese^32^. Moreover, the villi play a crucial role in the elimination of large particles, which allow only fine particles and fluid to be pushed from the colonic contents into the caeca by colonic antiperistalsis^33^.

Secretory IgA (SIgA) in avian is the dominant immunoglobulin in the external intestinal secretions, which constitutes the first-line immunological barrier against pathogens. The major roles of SIgA include neutralisation of microbial toxins and viruses and the prevention of microbial colonisation in the mucosal surfaces^34^. The present study showed that SIgA were scattered in the caecal lamina propria, and epithelial cells and the reaction was intense on the outer surface membrane suggesting an active transport of SIgA into the external secretions. These characteristic distributions in the caecal mucosa may be important for the prevention of intestinal antigen influx into internal body compartments.

The goblet cells appeared on the 13^th^ ED and were the other major cellular component in the epithelium of the caecum. The proximal part possessed more goblet cells than the other regions as in domestic chickens^35^. These cells are functionally significant by virtue of their capacity to secrete a sulphated mucoprotein complex, which acts as a lubricant and protective coating in the intestine^19^. Few enteroendocrine cells were also found in the surface epithelium and are characterised by their secretory granules and are thought to produce gastrin, glucagon, somatostatin, and bombesin^36^.

Lamina propria of the proximal part of the caecum was characterised by the high vascularity and rich cellular composition. Few smooth muscle cells from the underlying muscularis mucosa were extended into the core of the villi of the proximal part of the caecum causing the villi to become individually motile as observed in mammals^37^. The presence of the crypts of Lieberkuhn in the lamina propria-submucosa in this study indicated that some enzymatic breakdown of food particles might occur in the caeca.

The current study revealed that the CTs were demonstrated on the 13^th^ ED and became well-developed at two weeks post-hatching. More importance is attributed to the presence of CTs as structures similar to the mammalian Peyer’s patches, which are absent from the small intestines of birds. The present study showed that CD20-positive B-lymphocytes were immunolocalised in the CTs that indicating the involvement of CTs in cell-mediated immune functions^38^. In adult birds, the CTs constitute a major component of the avian gut-associated lymphatic tissue^39^. Studies indicated that CTs neutralise the antigens that enter the caeca due to the reflux of urates^16^. They might also represent a bursal equivalent^40^ and are suggested to play a critical role in the differentiation of stem cells into B lymphocytes^38^. In addition, diffuse lymphatic tissues including CD3-positive T lymphocytes were identified in the lamina propria throughout the caecal wall in both embryonic and post-hatching life. The occurrence of this diffused lymphatic tissue highlights the immunological surveillance against the caecal luminal contents and thus helps in the maintaining of the caecal microenvironment. Furthermore, T and B cells initiate a series of antigen-specific and nonspecific responses following infection^41^. A study of Kumary and colleagues^42^ recorded the presence of mast cells, plasma cells, erythrocytes, eosinophils and macrophages in the caecal wall of Japanese quails with heavy infiltration of lymphocytes.

Our study demonstrated the presence of intraepithelial lymphocytes (IELs) in the epithelial layer lining the caecum. IELs consist of highly specialised T and B lymphocytes^43^ and natural killer (NK) cells^44^. Our immunohistochemical results indicated that the majority of IELs are CD3-positive T lymphocytes, which are also identified within the lining epithelium of the oral cavity, the intestine, the reproductive tract, and the respiratory tract^34^. We suggested that their main function in the developing caecum is to maintain the mucosal barrier integrity through protecting the epithelium against pathogenic agents or immune‐induced pathology. In addition, IELs play a critical role in the mucosal immune system by performing a variety of regulatory functions, including cytokine production, cytotoxic activity, and induction of apoptosis in intestinal epithelial cells^45^.

Moreover, the present study revealed that the encapsulated CTs at pre-hatching periods were covered by columnar epithelial cells with goblet cells and few M-cells. While M-cells in chickens were observed only in two months old birds^46^. The M-cells were functionally characterised by active uptake of pathogens and antigens, such as horseradish peroxidase, ferritin, and latex particles^47^.

The present study revealed the presence of the dendritic cells (DCs) in quail caecum. They form a densely interconnected network in the submucosa. We suggest that they play a critical role in the induction of an immune response since they are antigen-presenting cells and their main function is the processing of antigen materials to present it to the T cell^48^. In chicken, a possible DC was first demonstrated in the bursa of Fabricius^49^ and the germinal centre of the CTs^50^. The dendritic cell in birds is characterised by its elongated shape with short dendrite-like processes^51^.

Rodlet cells were demonstrated in the current study for the first time amongst the surface epithelial cells of the quail caecum. The study by Abdalla and colleagues^52^ recently demonstrated the presence of rodlet cells in the colon and caecum of geese. The rodlet cells perform many functions; Reite^53^ reported them as non-specific immune cells while Reite, Evensen^54^ considered them as eosinophilic granulocytes. Moreover, Bielek^55^ supposed that the rodlet cells are migrating secretory cells.

The present results indicated that the tunica muscularis showed bundles of nerve fibres. Similar results were obtained in ducks by Mahdi^56^. This muscular layer showed a marked increase in thickness at post-hatching that may increase the capacity of the caeca to return the particles back to the colon.

Telocytes were identified in the quail cecum for the first time in this study. They consist of a spindle-shaped cell body with two long telopodes that made up frequent close contacts with neighbouring smooth muscle cells. Telocytes (TCs) are a novel type of interstitial cells that have been identified recently in various organs of humans and laboratory mammals^57^, however, ultrastructural identification of TCs remains unclear in birds. In the present study, S-100 protein was expressed in TCs of the caecal lamina propria, suggesting a neuronal property of these cells^58^. Also, S-100 protein positive-TCs were recorded between the muscle layers of the caecum suggesting their role in contractility. Furthermore, expression of S-100 proteins in the caecum may represent its roles in the maintenance of Ca^+2^ homeostasis, cell cycle progression, cellular structure, as well as modulation of enzyme activities^59^. Many functions have been suggested to telocytes, including a stromal organisation through alteration of intercellular communication. Taken together, the TC could be involved in the secretion, contractility and immune surveillance^60^.

## Conclusion

The study provides the descriptions of the caecal development in Japanese quail with an organisation of its cellular and stromal elements. Different immune cell types were identified in the caecum based on their morphological features. The rodlet cells, lymphocytes, dendritic cells, macrophages, and different types of leukocytes were identified in the mucosa and submucosa of the caecum in the Japanese quail. The current study is the first one to focus on the distribution of telocytes in the quail caecum. These findings are not only presenting fundamental data on the development of the caecum but also highlight on a number of questions: what the accurate function of telocytes in quail caecum and why the rodlet cells display this distribution?

### Ethical approval and consent to participate

The study was approved by the Ethics Committee of Assiut University, Egypt.
